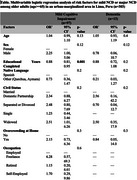# Risk factors associated with neurocognitive disorder and depression among adults from an urban‐marginalized area of Lima, Peru

**DOI:** 10.1002/alz70860_101750

**Published:** 2025-12-23

**Authors:** Monica M Diaz, Eder Herrera‐Perez, Nilton Custodio, Rosa Montesinos, Chhitij Tiwari, Serggio Lanata

**Affiliations:** ^1^ University of North Carolina at Chapel Hill School of Medicine, Chapel Hill, NC, USA; ^2^ Grupo de investigación Molident, Universidad San Ignacio de Loyola, Lima, Peru; ^3^ Unidad de Investigación de Deterioro Cognitivo y Prevención de Demencia, Instituto Peruano de Neurociencias, Lima, Lima, Peru; ^4^ Universidad de San Martín de Porres, Facultad de Medicina, Centro de Investigación del Envejecimiento, Lima, Lima, Peru; ^5^ Instituto Peruano de Neurociencias, Lima, Lima, Peru; ^6^ University of North Carolina at Chapel Hill, Chapel Hill, NC, USA; ^7^ Memory and Aging Center, UCSF Weill Institute for Neurosciences, University of California, San Francisco, San Francisco, CA, USA; ^8^ Global Brain Health Institute, San Francisco, CA, USA

## Abstract

**Background:**

NCDs and depression pose substantial public health challenges, particularly in urban‐marginalized areas of low‐to‐middle‐income countries (LMICs), where socioeconomic disparities amplify mental health concerns. The risk factors for NCDs among both younger and older adults in urban‐marginalized areas of Latin America have been inadequately studied. Our study investigates the prevalence and risk factors of neurocognitive disorders (NCDs) and depression among community‐dwelling younger and older adults in Puente Piedra, an urban‐marginalized district of Lima, Peru.

**Method:**

A population‐based study was conducted from July through September 2022, incorporating door‐to‐door visits and structured questionnaires to collect demographic, health, and socioeconomic data from 900 community‐dwelling adults aged 30 years and older. Neurocognitive assessments included the Addenbrooke's Cognitive Examination for younger adults, Rowland Universal Dementia Assessment Scale for older adults, and Patient Health Questionnaire (PHQ‐9) for depression screening.

**Result:**

We found that 75.6% of participants were cognitively normal, 20.1% had possible NCD without functional impairment, and 4.2% with functional impairment. Additionally, 40% were depressed. After controlling for covariates, lower educational levels and lower socioeconomic levels were risk factors for NCD among older adults. Among younger adults, lower educational levels, a native language other than Spanish, hypertension, and depression were risk factors for NCD. Risk factors for depression included female sex, lower educational level, a native language other than Spanish, overcrowding in the home, and a history of chronic diseases.

**Conclusion:**

Our results highlight that potentially modifiable risk factors, such as hypertension, educational level, and depression may play a role in NCDs among residents of an urban‐marginalized area of Peru. These findings underscore the critical need for targeted interventions and policies to address mental health and healthcare disparities in urban‐marginalized areas of LMICs.